# Imaging-based assessment of corneal graft rejection after penetrating keratoplasty: insights from confocal microscopy and anterior chamber analysis

**DOI:** 10.3389/fmed.2025.1696949

**Published:** 2025-12-15

**Authors:** Yuan Lin, Miaomiao Liu, Hanqiao Li, Xie Fang, Zhiwen Xie, Shunrong Luo, Xianwen Xiao, Huping Wu

**Affiliations:** 1 Xiamen Eye Center and Eye Institute of Xiamen University, School of Medicine, Xiamen, China; 2 Xiamen Clinical Research Center for Eye Diseases, Xiamen, Fujian, China; 3 Xiamen Key Laboratory of Ophthalmology, Xiamen, Fujian, China; 4 Fujian Key Laboratory of Corneal & Ocular Surface Diseases, Xiamen, Fujian, China; 5 Xiamen Key Laboratory of Corneal & Ocular Surface Diseases, Xiamen, Fujian, China; 6 Translational Medicine Institute of Xiamen Eye Center of Xiamen University, Xiamen, Fujian, China

**Keywords:** penetrating keratoplasty, corneal graft rejection, primary disease, visual outcome, risk factor

## Abstract

**Objective:**

This study aimed to explore the distribution of rejection and visual changes after optical and therapeutic penetrating keratoplasty (PKP) in Southeastern China.

**Methods:**

This retrospective study included 104 patients who underwent either optical or therapeutic PKP between December 2014 and April 2022 at the Xiamen Eye Center. The collected data included demographic characteristics, primary disease, visual acuity, *in vivo* confocal microscopy (IVCM) findings, and postoperative outcomes. Endothelial inflammation was assessed using IVCM, and anterior chamber paracentesis was performed on patients with recurrent rejection (>2 hospitalizations) for viral and cytokine analyses.

**Results:**

Of 104 eyes (68 men and 36 women; mean age 51.9 ± 12.9 years), 42 received optical PKP and 62 received therapeutic PKP. The mean host bed size was 7.81 ± 0.75 mm, and the graft size was 8.28 ± 0.75 mm. During follow-up, four patients developed secondary glaucoma and one relapsed. A total of 12 patients (2 optical, 10 therapeutic) required repeat PKP and were successfully managed. A larger graft size (>7.8 mm) and therapeutic PKP were identified as significant risk factors for graft rejection, whereas conjunctival congestion, infiltration depth, and femtosecond-assisted PKP were not significantly associated with rejection. Among the 40 cases with recurrent rejection, aqueous fluid analysis revealed viral infection in 20% (3 VZV, 10 HSV, and 7 CMV). Elevated levels of inflammatory cytokines were found in 32 cases, and they responded positively to steroid treatment. However, three cases showed a poor response to the treatment.

**Conclusion:**

PKP effectively addresses both optical and therapeutic indications; however, larger grafts and therapeutic PKP increase the risk of rejection. Imaging with IVCM and anterior chamber analysis provides valuable diagnostic and prognostic information, supporting individualized management of graft rejection.

## Introduction

Severe corneal diseases can lead to irreversible vision loss or may necessitate ocular evisceration. Corneal transplantation is a crucial therapeutic procedure used to restore the structure and function of the cornea in patients (1). For patients with severe visual impairment or corneal tissue perforation, penetrating keratoplasty (PKP) remains the treatment option for severe corneal disease in ocular surface patients without lamellar grafting or endothelial transplantation surgery (2). Indications for PKP include bullous keratopathy, keratoconus, corneal scars, corneal perforation secondary to infection, and Fuchs endothelial dystrophy (3, 4). Therefore, the purpose of performing PKP in patients can be divided into two main areas: optical and therapeutic. It aims to address long-term corneal damage or scarring and restore corneal transparency for improved vision (5, 6). Infectious keratitis can be caused by bacteria, fungi, viruses, and other infections, such as amoebas. These infections can lead to rapid or progressive erosion of the corneal structure (7). When corneal perforation occurs, PKP is the only surgical procedure that can reconstruct the structure of the ocular surface (8).

As a result of corneal immune privilege, corneal transplantation has a reasonably high success rate compared to other organ transplants (9). The cornea’s immune privilege is not absolute, and any disruption could lead to activation of the antigen-host immune system in the donor corneal button, causing immune rejection (10). Corneal rejection after PKP is the primary postoperative complication, with a higher likelihood of rejection compared to lamellar or endothelial transplantation (11). Previous research indicates that limbal neovascularization, larger graft size, prior ocular surgery, and the use of glaucoma medication are risk factors for post-PKP rejection (12). Continuous rejection, whether persistent or chronic, triggers inflammation that degrades graft collagen, leading to graft melting and an increased risk of perforation (13). Limited information is available on comparisons between optical and therapeutic PKP corneal rejection and subsequent graft melt. Therefore, accurate and early identification of graft rejection remains crucial for improving postoperative outcomes after PKP. Advanced imaging techniques, including *in vivo* confocal microscopy (IVCM) and anterior segment optical coherence tomography (AS-OCT), provide valuable insights into cellular and structural changes during graft rejection, enabling more objective and quantitative evaluation than conventional slit-lamp examination.

This study involved retrospective observation of 104 patients who underwent PKP surgery. This study aimed to comprehensively evaluate corneal graft rejection following PKP using multimodal imaging techniques. Specifically, we analyzed the morphological characteristics of the graft–host interface using IVCM and quantified the anterior chamber inflammatory activity using AS-OCT. Furthermore, we compared optical and therapeutic PKP indications to identify potential clinical and imaging predictors associated with graft rejection. Our findings may help establish a more standardized imaging-based assessment for postoperative monitoring and early diagnosis of graft rejection. We conducted a retrospective analysis of the primary cause of PKP and examined the visual changes in patients undergoing this procedure. Furthermore, we identified the risk factors for graft dissolution following rejection. These findings offer valuable insights into the use optical and therapeutic PKP, improving the quality of life regarding eye health, and preventing blindness.

## Methods

### Patients

The study adhered to the ethical guidelines of the Declaration of Helsinki. The Human Ethics Committee of Xiamen University, affiliated with the Xiamen Eye Center, thoroughly reviewed and approved the study involving human participants. Before surgery, all patients provided written informed consent, granting permission to use their data for future teaching and research at the institution. Clinical data included information from 104 patients, each with one eye involved, collected from December 2014 to April 2022. There were 68 male and 36 female patients, aged between 47 and 84, with a mean age of 51.90 ± 12.89 years. All patients who underwent PKP were evaluated for corneal lesions and ocular surface conditions and were deemed unsuitable for other surgical options, including lamellar keratoplasty and endothelial keratoplasty. All patients underwent PKP for the first time.

### Diagnosis, inclusion, and exclusion criteria

#### Optical indications

The goal is to enhance vision by replacing the cloudy cornea with a clear donor cornea. This procedure is suitable for patients who were only eligible for PK, including but not limited to those with vision-impairing corneal scars. Other common causes include keratoconus, corneal dystrophy, degeneration, and scars caused by trauma or keratitis.

#### Therapeutic indications

The purpose of surgery was to restore or maintain the anatomy of the cornea. Severe structural pathology includes stromal thinning and descemetocele, along with surgery following the removal of inflammatory corneal tissue that is unresponsive to medications.

#### Diagnosis of Graft rejection

The diagnosis includes typical history, clinical manifestations, and laboratory tests. The patient’s eye condition was assessed, the medical history was collected, and the affected corneal area was examined as part of the diagnosis. Evidence of corneal infiltration, hyperplasia, or ulceration was also obtained. Clinical signs of graft rejection include corneal edema, keratic precipitates on the corneal graft but not on the peripheral recipient cornea, corneal vascularization, stromal infiltrates, a Khodadoust line, an epithelial rejection line, and subepithelial infiltrates. The Khodadoust line separates the immunologically damaged endothelium from the unaffected endothelium. In the damaged area, the endothelium decompensates, resulting in stromal and epithelial edema.

#### Inclusion criteria

Ulcer cases were included in the therapeutic PKP group when medical therapy failed to control the infection or when corneal thinning and perforation risk necessitated urgent full-thickness transplantation to preserve the structural integrity of the ocular surface. Patients who met the diagnostic criteria for corneal graft rejection during follow-up, confirmed by clinical examination and ancillary testing when necessary, and those who had complete demographic, preoperative, surgical, and follow-up data were included.

#### Exclusion criteria

Patients with coexisting ocular surface diseases, including neurotrophic corneal ulcers, chronic graft-versus-host disease, conjunctival keratosis, or exposed corneal lesions, were excluded. Patients with poor general health and an inability to tolerate surgery, those with severe dry eye or incomplete eyelid closure, those with severe allergic constitution or allergies to test materials, and those with a single eye or vision loss in the contralateral eye were also excluded.

### Clinical evaluation

All patients underwent a thorough systemic assessment, and detailed clinical information was recorded preoperatively (Table 1). All experimental data and imaging features were obtained at disease onset. All patients underwent a comprehensive examination of their systemic disease history, including onset and recovery time. The examination included best-corrected visual acuity (BCVA) measurement, intraocular pressure (IOP) measurement, and *in vivo* confocal microscopy. The BCVA measurement was conducted using the Snellen chart. The results were analyzed statistically and converted to Logarithm of Minimum Angle of Resolution (LogMAR) units (14). For counting fingers or worse, the following transformations were used: counting fingers, 2.0 LogMAR; hand movement, 2.3 LogMAR; light perception, 2.6 LogMAR; no light perception, 2.9 LogMAR. A retrospective chart review was conducted for all subjects, with relevant case details such as demographics, clinical characteristics, underlying infections, and treatment modalities transcribed in a standardized format.

**Table 1:** Stepwise evaluation process of patients undergoing penetrating keratoplasty.

Step	Assessment item	Description/Purpose
1. Medical and Ocular History Collection	Systemic and ocular history	Record trauma, infection, previous ocular surgery, systemic diseases (e.g., diabetes, hypertension, autoimmune disorders), and medication use.
2. Baseline Ophthalmic Examination	Visual acuity	Measure best-corrected visual acuity (BCVA) using the Snellen chart and convert to LogMAR for analysis.
	Intraocular pressure (IOP)	Assess using non-contact or applanation tonometry.
	Slit-lamp biomicroscopy	Examine ocular surface, corneal transparency, infiltrates, epithelial defects, ulcers, stromal thinning, neovascularization, and Descemet’s folds.
	Fundus examination	Evaluate the posterior segment if corneal clarity permits.
3. Corneal Imaging and Structural Evaluation	AS-OCT	Determine the depth and extent of corneal lesions and involvement of Descemet’s membrane and endothelium.
	IVCM	Assess epithelial cell integrity, stromal inflammatory infiltration, and endothelial morphology.
	CFS	Detect epithelial defects and grade corneal damage according to the Oxford scale.
4. Etiological Investigation	Microbiological and molecular tests	Collect corneal scrapings for Gram/fungal staining, bacterial and fungal culture, and PCR testing for HSV, VZV, and CMV when indicated.
5. Classification of Indications	Type of PKP	Optical PKP: for corneal opacity, endothelial decompensation, or ectatic disorders. Therapeutic PKP: for active infection, perforation, or impending rupture refractory to medical therapy.
6. Preoperative Medical Optimization	Infection and inflammation control	Administer topical/systemic antimicrobial therapy for infection and corticosteroids or immunomodulators for inflammation before surgery.
	Systemic evaluation	Obtain systemic assessment and anesthetic clearance prior to operation.

During each visit, visual acuity, IOP, and slit lamp examinations were performed to assess the postoperative ocular surface, examine the corneal graft, and observe signs of epithelial repair, edema resolution, or any deterioration. Subjective and objective symptoms at the final postoperative follow-up were recorded, including corneal epithelial healing, neovascularization, and infection control. “Cure” was defined as the absence of ulcer progression, and either scarred or epithelialized; “improvement” was defined as control of irritation or complications after conservative treatment; “failure” was defined as a second surgery, including corneal transplantation, conjunctival flap cover, or enucleation. Once signs of disease remission became apparent, local immunosuppressive eye drops were gradually reduced over the next 2–3 weeks and then stopped completely. All patients were monitored for evidence of recurrence for at least 3 months after the discontinuation of medication. Follow-up appointments were scheduled on postoperative day 7, week 2, and months 1 and 3, and thereafter every 3 months depending on the clinical condition. Immunologic graft failure was diagnosed if signs of rejection were not clear within 2 months of treatment.

#### Clinical evaluation indications

A slit-lamp microscope (BQ900IM900) and its photographic device (Haag-Streit, Switzerland) were used to capture images of the front part of the eye.

#### Invasive lesion scale score

Diffusion distribution (0 points); ulcer area size was less than 1 / 3 cornea (1 point); ulcer area size was 1/3 ~ 2/3 cornea (2 points); ulcer area size was 2 / 3–1 cornea (3 points).

#### Conjunctival congestion score

Zero points, no congestion; 1 point, single-vessel congestion; 2 points, mild diffuse congestion; 3 points, severe local congestion; 4 points, severe diffuse congestion.

### Corneal fluorescein staining (CFS)

The patient’s ocular surface was soaked in fluorescein solution. Then, the patient blinked several times; after 3 min, CFS was evaluated using a slit-lamp microscope illuminated with cobalt blue to evaluate localized corneal and conjunctival epithelial desiccation areas. Staining was recorded using a modified Oxford grading scheme (15).

### AS-OCT image

AS-OCT images were captured using the Ophthalmic Optical Coherence Tomography System (RTVue XR, Optovue Inc., USA) to determine the depth of invasion. The OCT images of the participants were captured by the same operator using the same machine. The “Line” scan pattern was selected for scanning. Measurements were taken at the central point of the lesion. The exact location and protocol were maintained during the last follow-up for measurement acquisition. The corneal specialist who performed the measurements verified the infrared images provided by the AS-OCT device to ensure consistency with the locations used during the final follow-up.

AS-OCT measured the depth of lesion invasion score: diffusion distribution (0 points); ulcer invasive scale was less than 1/3 (1 point); ulcer invasive scale was 1/3 ~ 2/3 (2 points); ulcer invasive scale was 2/3–1 (3 points).

### IVCM and anterior chamber analysis

The IVCM (HRT III/Rostock) was used for corneal examination. Before imaging, 0.5% proparacaine was applied three times to the conjunctival capsule, and a gel (Alcon, Fort Worth, TX, USA) was inserted between the lens surface (composed of poly(methyl methacrylate) (PMMA) or poly(methyl methacrylate-co-propyl methacrylate) (PMMP)) and the lens contact cap to act as the imaging medium. A 670 nm semiconductor laser served as the excitation light source, with manual rotation adjustments made to ensure a clear image. The objective was a 60x intrusive lens with 800x magnification, covering a scan area of 400um^2^ and a picture pixel of 384 × 384. The central cornea was used as a reference point, and various layers of the cornea, including the corneal epithelial layer, Bowman’s layer, stroma, Descemet’s membrane, and endothelial layer, were examined vertically.

Endothelial inflammation of the corneal grafts was assessed using confocal microscopy, and anterior chamber paracentesis was performed in patients with recurrent graft rejection (hospitalized >2 times).

### Treatment strategies Procedure of the classic and femtosecond laser-assisted penetrating keratoplasty procedure

The patient was placed in the supine position. The anesthesiologist completed anesthesia (a general anesthesia protocol that included remimazolam powder, fentanyl injection, propofol medium/long-chain fat emulsion injection, cisatracurium injection, dezocine injection, atropine injection, and ondansetron injection), disinfectant (5% povidone-iodine), and eyelid. The corneal trephination of the host bed was drilled with a negative pressure ring in the center of the cornea, with a diameter of 6.75 ~ 11.00 mm. Subsequently, 0.5 mL of viscoelastic was injected into the anterior chamber. Completion of the implantation was assisted by corneal scissors, and the pupil, iris, and crystalline structures were observed. The donor cornea was preserved in Optisol corneal storage medium before surgery. Under the protection of an artificial anterior chamber system, donor corneal trephination was performed with the epithelium facing downward, using a trephine diameter ranging from 7.25 to 11.50 mm, and the graft was then positioned onto the recipient bed.

If the donor graft was prepared using femtosecond laser assistance, the following parameters were applied: the cutting diameter was adjusted according to the patient’s corneal size (7.25–11.50 mm), the cutting depth was set to 1,000 μm, the flap-edge cutting angle to 90°, and the laser energy to 2.0 μJ. The cornea was fixed within the artificial anterior chamber, and the femtosecond laser created a vertical trephination ring from the epithelial side. The prepared graft was subsequently transferred and precisely placed on the host bed.

The donor graft and host bed were sutured with 10–0 nylon using interrupted stitches placed at approximately four-fifths of the corneal thickness, ensuring moderate tension and burying of the knots within the deep stromal layer. The residual viscoelastic material in the anterior chamber was carefully replaced with a balanced salt solution to ensure a well-formed anterior chamber and clear aqueous and watertight wound closure. Sterile ointment and an eye pad were applied as ocular dressings. After recovery from general anesthesia, the patient was transferred to the ward and received routine postoperative anti-inflammatory and anti-rejection prophylactic medication.

### Statistical analysis

All data were entered into an Excel spreadsheet and analyzed using the Statistical Package for the Social Sciences (SPSS), version 25 (IBM, Chicago, Illinois, USA). Continuous data are presented as mean ± SD for normally distributed data. As appropriate, patient-related observations were compared with either the one-way ANOVA test or the chi-squared test. MedCalc version 22.007 analyzed the optimal intercept value of clinical indicators and the K-M analysis for the risk of repeat PKP. A generalized estimating equation approach to observations nested within the same individual was used to compare eye outcomes. All tests were two-tailed, and a *p*-value of <0.05 was considered statistically significant.

## Result

There were 104 eyes, including 68 males (65.38%) and 34 (34.62%) females, with a mean age of 51.90 ± 12.89 years (47 to 84 years). They received optical PKP in 42 patients and therapeutic PKP in 62 patients. A total of 39 (37.50%) patients had a history of trauma. Among all patients, the most common etiology was fungal infection, with 33 cases (31.73%), followed by corneal leukoplakia in 24 (23.07%) (17 cases involved scarring following viral infection), corneal endothelial decompensation in 15 cases (7 were Fuchs’ dystrophy) (14.42%), sterile ulcer in 14 cases (14.46%), corneal perforation caused by viruses in 9 cases (8.65%), bacteria in 4 cases (3.84%), keratoconus in 3 cases (2.87%), mixed infection in 1 case (0.98%), and graft-versus-host disease in 1 case (0.98%). The indications for optical and therapeutic PKP in this cohort are summarized in Table 2.

**Table 2:** Primary indications for optical and therapeutic penetrating keratoplasty.

Primary disease	Optical PKP (*n*, %)	Therapeutic PKP (*n*, %)	Total (*n*, %)
Fungal keratitis	0 (0%)	33 (31.7%)	33 (31.7%)
Viral keratitis/Post-viral scar	17 (40.5%)	9 (8.6%)	26 (25.0%)
Corneal endothelial decompensation	15 (35.7%)	0 (0%)	15 (14.4%)
Sterile ulcer	0 (0%)	14 (13.5%)	14 (13.5%)
Corneal perforation (bacterial)	0 (0%)	4 (3.8%)	4 (3.8%)
Keratoconus	3 (7.1%)	0 (0%)	3 (2.9%)
Mixed infection	0 (0%)	1 (1.0%)	1 (1.0%)
Graft-versus-host disease	0 (0%)	1 (1.0%)	1 (1.0%)
Total	42 (40.4%)	62 (59.6%)	104 (100%)

The top three locations of lesion distribution were the central cornea in 41 cases (39.42%), the lower central cornea in 32 cases (30.76%), and the entire cornea in 8 cases (7.69%). Others included four patients with diabetes and three hypertensive patients. The size of the PKP graft bed was 7.81 ± 0.75 mm, and the graft size was 8.28 ± 0.75 mm. The details of the optical and treatment groups are shown in Table 3. Preoperative BCVA was 2.00 ± 0.47 LogMAR, postoperative BCVA 1.38 ± 0.61 LogMAR, and BCVA 1.63 ± 0.67 LogMAR at the time rejection occurred. Visual performance improved after PKP, and different degrees of visual loss were observed when rejection occurred. Preoperative optical PKP was BCVA 1.90 ± 0.48 LogMAR, postoperative BCVA 1.27 ± 0.58 LogMAR, and BCVA was 1.47 ± 0.66 LogMAR at the time of rejection occurrence. Therapeutic PKP had preoperative BCVA 2.07 ± 0.45 LogMAR, postoperative BCVA 1.45 ± 0.63 LogMAR, and BCVA 1.73 ± 0.66 LogMAR at the time of rejection onset. There was no significant difference in BCVA between the optical and therapeutic PKP groups (*p* = 0.392, *p* = 0.189, and *p* = 0.960, respectively). In addition, patients without femtosecond laser-assisted PKP had BCVA 2.03 ± 0.44 LogMAR, BCVA 1.38 ± 0.60 LogMAR, and BCVA 1.65 ± 0.65 LogMAR at the time of rejection. Patients with femtosecond laser-assisted PKP had BCVA 1.88 ± 0.58 LogMAR, BCVA 1.37 ± 0.68 LogMAR, and BCVA 1.50 ± 0.77 LogMAR at the time of rejection. There was no significant difference in BCVA between the groups with or without femtosecond laser-assisted PKP (*p* = 0.247, *p* = 0.916, and *p* = 0.406, respectively).

**Table 3:** Demographic, clinical, and treatment information of patients.

	Optical PKP	Therapeutic PKP	*p*-value
Sex			0.073†
Male, *n* (%)	10(40%)	45(64%)	
Female, *n* (%)	15(60%)	17(36%)	
Age, years	49.10 ± 15.27	53.73 ± 10.81	0.073†
Symptom duration, months	85.32 ± 113.70	2.05 ± 6.26	<0.001†
BCVA, logMAR	1.90 ± 0.48	2.07 ± 0.45	0.626†
IOP, mmHg	13.12 ± 5.64	12.70 ± 8.07	0.774†
Congestion score	0.44 ± 0.67	2.54 ± 0.71	<0.001†
Ulcer invasive scale, diameter in mm	4.49 ± 3.52	5.86 ± 1.74	
Ulcer invasive scale score	1.90 ± 0.88	2.06 ± 0.53	0.251†
Depth of Invasion score	1.39 ± 1.39	2.43 ± 0.92	<0.001†
Planting bed diameter, in mm	7.44 ± 0.31	8.05 ± 0.85	<0.001†
Graft size, mm	7.93 ± 0.30	8.5 ± 0.86	<0.001†
Femtosecond laser-assisted			0.005‡
Yes	12 (28.57%)	5 (8.06%)	
No	30 (71.43%)	57 (91.94%)	

^†^One-way ANOVA test. ^‡^Chi-squared test.

**Figure 1:**
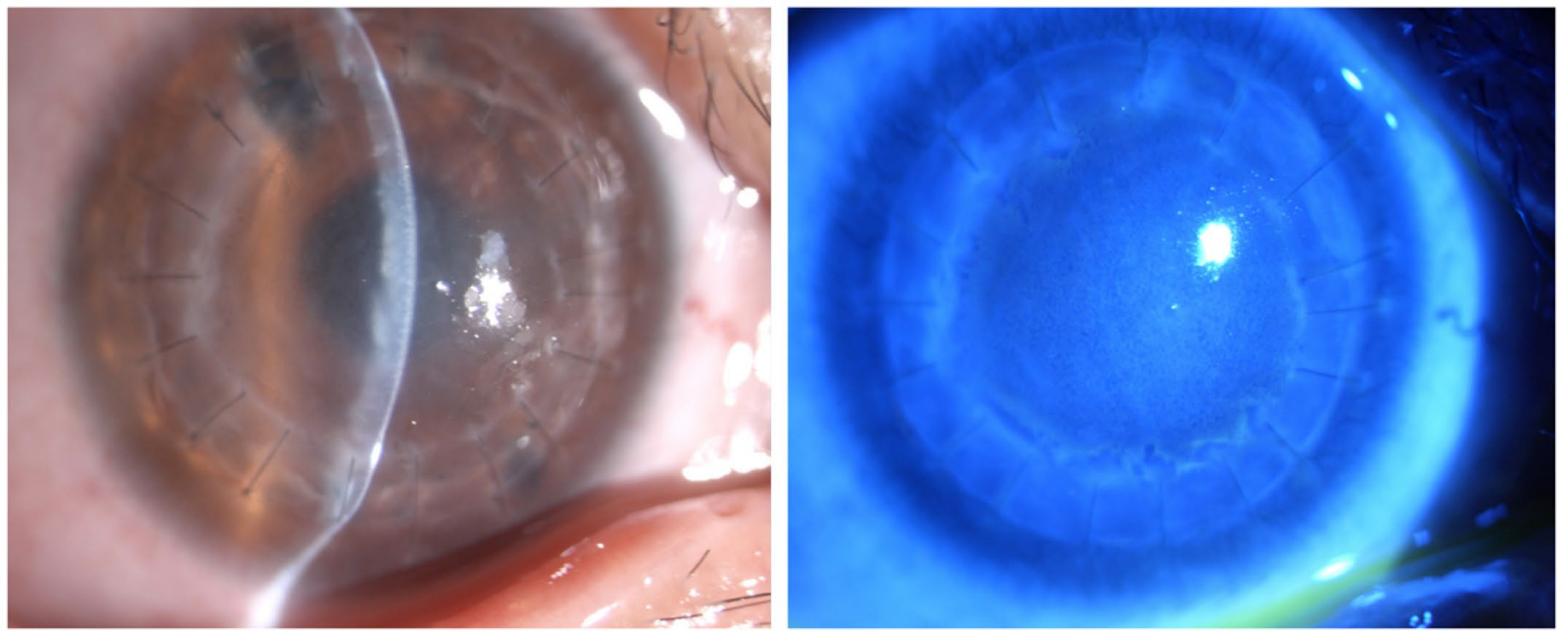
Slit lamp image features of rejection after PKP. The slit lamp showed corneal edema, corneal precipitation on the corneal graft, infiltration of the stroma, an epithelial rejection line, and subepithelial infiltration.

**Figure 2:**
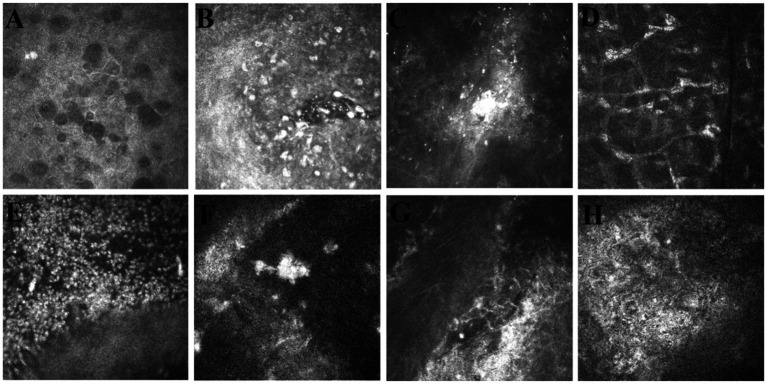
IVCM image features of postoperative rejection after PKP. **(A)** Bubble changes in the epithelium and between layers. **(B)** Epithelial layer epithelial defect with inflammatory cell infiltration. **(C)** Focal reflectivity in a shallow matrix. **(D)** Bubble-like appearance of enlarged stromal cell space in the medium and deep matrix layers. **(E)** Mass inflammatory cell attachment to the endothelium. **(F)** Calm after a focal, highly reflective cornea in the endothelium. **(G)** Endothelial surface rupture and high reflectivity. **(H)** Endothelial cells lose their typical morphology with unclear borders, and the black color continues to change.

Slit lamp photographs revealed epithelium with a disseminated epithelial defect, matrix edema, and folds of the posterior elastic layer (Figure 1). IVCM imaging features indicated chronic vesicular changes and inflammatory infiltration of epithelial defects in the epithelial layer, mixed with focal highlights and scar changes, enlarged space, varying inflammation of the endothelium, adhesion of corneal aging, and rupture-like changes in the endothelial layer (Figure 2). In patients with recurrent rejection (n = 40), anterior chamber fluid analysis revealed viral infection in 20.0% (3 VZV-positive, 10 HSV-positive, and 7 CMV-positive). The remaining patients tested negative for viral nucleic acids and antibodies. Elevated inflammatory cytokine levels were observed in 32 patients, showing a marked response to corticosteroid therapy. In three cases, cytokine levels decreased, and hormone therapy achieved only moderate effects.

**Figure 3:**
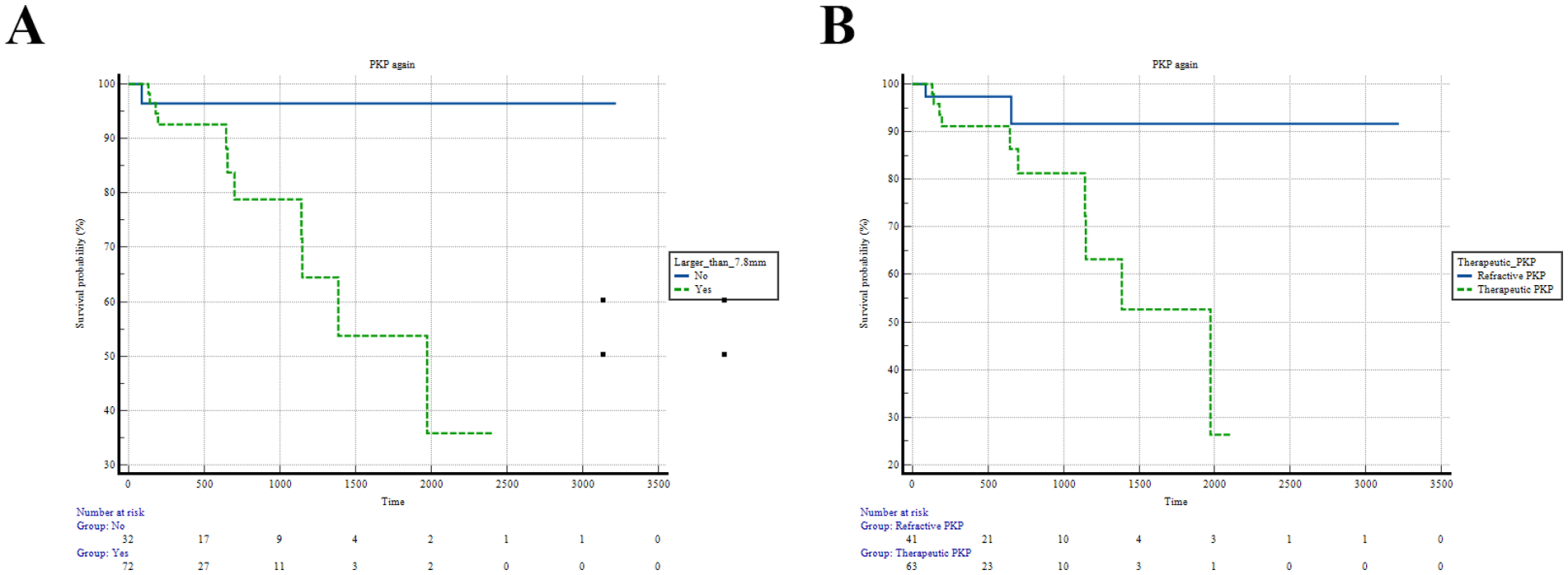
Risk factors for PKP after rejection. **(A)** Greater than 7.8 mm has a higher risk (*p* = 0.036). **(B)** Therapeutic PKP has a higher risk than optical PKP (*p* = 0.037).

During the follow-up, four patients developed secondary glaucoma, and one patient had a recurrence. Finally, 12 cases (2 optical and 10 therapeutic) were controlled after repeated PKP. K-M survival analysis (Log-Rank) suggested that graft size> 7.8 mm (*p* = 0.036) and therapeutic PKP (*p* = 0.037) were risk factors for rePKP due to postoperative rejection (Figure 3). However, conjunctival congestion score (*p* = 0.306), lesion distribution (*p* = 0.769), depth of infiltration (*p* = 0.396), and femtosecond laser-assisted PKP (*p* = 0.488) were not considered risk factors for further PKP.

## Discussion

PKP is used to manage a wide spectrum of corneal disorders. However, corneal graft rejection remains one of the most common postoperative complications (16). Corneal rejection is primarily a cell-mediated immune response that can cause progressive endothelial damage and loss of graft clarity (17). Persistent or recurrent rejection may eventually lead to graft melting, necessitating repeat PKP procedures (18). In this study, patients who underwent therapeutic PKP for corneal ulcers were identified as a high-risk group for postoperative rejection, emphasizing the importance of adequate preoperative medical management to control infection and inflammation before transplantation. Our results demonstrated that both optical and therapeutic PKP effectively improved postoperative visual acuity and restored corneal integrity. However, femtosecond laser assistance did not yield a significant advantage in visual recovery in this cohort.

In addition to these clinical and immunological characteristics, our findings indicate that integrating structural and cellular imaging may provide a more refined framework for the early surveillance of rejection. IVCM allows real-time visualization of keratocyte activation, dendritic cell density, and endothelial cell morphology, whereas anterior segment OCT offers quantitative assessment of corneal thickness and graft–host junction integrity. The complementary information obtained from these modalities enables clinicians to identify subtle inflammatory changes before overt signs, such as edema or keratic precipitates, become clinically apparent. This multimodal imaging approach is particularly advantageous for patients with limited symptom awareness or preexisting corneal opacities that hinder slit-lamp evaluation. Establishing standardized imaging-based thresholds for inflammatory cell density or microstructural alterations may enhance early diagnostic accuracy and support the timely escalation of immunosuppressive therapy, ultimately reducing the risk of irreversible graft failure.

We further observed that post-therapeutic PKP rejection occurred more frequently in patients with fungal keratitis, whereas post-optical PKP rejection was more common in eyes with corneal scarring secondary to viral infection. Previous studies have shown that herpes simplex virus type 1 (HSV-1) can induce lymphangiogenesis within the cornea through a mechanism independent of toll-like receptors, thereby compromising corneal immune privilege and predisposing the graft to immune rejection (19, 20). Moreover, PKP itself may represent a risk factor for HSV-1 transmission, as latent donor-derived HSV-1 strains can be reactivated in the recipient cornea following transplantation (21).

Patients with endothelial failure may not have been promptly diagnosed if they experienced graft rejection. Corneal graft rejection and inflammation are crucial in graft failure (22). Abnormal growth of new blood vessels in the limbus before and after surgery can cause inflammation and immune responses in the corneal epithelium, stroma, and endothelial cell layers. These inflammatory effects can significantly impact corneal transplantation before or after the procedure. When persistent inflammation and immune factors affect the graft, the response outcome may be uncertain, leading to potential rejection and graft inactivation (23). After examining patients who experienced rejection following PKP, we found that visual loss resulting from rejection was the main reason patients became aware of the problem. IVCM images showed consistent bullous changes in the corneal graft and subepithelial infiltration under the slit lamp. IVCM revealed inflammatory changes in different layers of the cornea (24). We believe that rejection following PKP may result from a vicious circle involving the activation of immune inflammation, tissue repair, and functional decompensation.

In a subset of recurrent rejection cases, aqueous humor analysis indicated the involvement of viral reactivation, particularly HSV, VZV, and CMV, which may trigger persistent inflammation despite conventional immunosuppression. The detection of elevated inflammatory cytokine levels in most patients further supports the concept that graft rejection is a multifactorial process in which immune dysregulation and latent viral activity intersect. Together, these observations underscore the value of combining IVCM imaging with anterior chamber fluid analysis to differentiate infectious from immune-mediated rejection and guide individualized management strategies.

The main subjective sign of rejection is loss of vision, and other symptoms may not be present. When rejection occurs, immune cells infiltrate the corneal surface, leading to increased inflammation in the eye. Rejection involves donor tissue, and various factors can influence it (25). Infection, surgery, and trauma can trigger the immune system to release cytokines, cell adhesion molecules, and growth and angiogenic factors that can invade the cornea and cause rejection. This process can also lead to neoangiogenesis and lymphangiogenesis, triggering immune activation and graft rejection (26).

Rejection of a transplanted graft is a complicated immune response. During this process, the recipient’s immune system identifies and reacts to foreign antigens in the transplanted cornea, thereby triggering an immune response against the graft. Each layer of the cornea, including the epithelium, stroma, and endothelium, can lead to rejection (27). The immunogenicity of different layers of the cornea can vary. Epithelial and stromal cells have less immune privilege compared to endothelial cells. Based on the endothelial response shown by IVCM, we hypothesize that immune rejection in the endothelium triggers an inflammatory response, leading to the exhaustion of endothelial cells and eventually causing endothelial decompensation. The ultimate failure of the cornea seems to be due to graft failure, possibly because surgical intervention disrupts corneal homeostasis and the blood-eye barrier, leading to a temporary increase in pro-inflammatory cytokines and creating a pro-apoptotic environment (28–30). It has been suggested that donor dendritic cells (DCs) can directly present antigens to draining lymph nodes through the direct pathway, whereas the indirect pathway involves host antigen-presenting cells processing donor antigens before presentation (31). The direct pathway appears to play a particularly important role in high-risk corneal beds with increased immunogenicity and loss of immune privilege (32). Experimental studies have demonstrated that JAK2 knockout leads to the deactivation of CD4^+^ T cells and reduced IFN-*γ* expression, thereby inhibiting DC development, maturation, and cytokine secretion, ultimately decreasing the incidence of graft rejection (33). Compared with stable allografts, acute rejection has been associated with altered B-cell maturation and a reduced frequency of regulatory CD4^+^ T cells, highlighting the complex immune dysregulation underlying rejection episodes. However, genetic or cellular assays based on peripheral blood are currently insufficiently sensitive for the clinical prediction or diagnosis of corneal rejection (34). Disruption of the corneal immune microenvironment and subsequent neovascularization can further intensify inflammation and immune activation, leading to corneal opacity, vision loss, and a self-perpetuating cycle of tissue damage (35).

The most common reasons for repeat corneal transplant surgery include intraocular lens-related issues, graft failure, Fuchs’ dystrophy, aphakic bullous keratopathy, keratoconus, and herpes keratitis. Chronic endothelial cell loss is the primary cause of graft failure in PKP recipients (36–38). This study found that grafts larger than 7.8 mm and therapeutic PKP were risk factors for repeat PKP. Identifying the risk factors for each type of corneal transplantation, such as deep matrix vascularization, history of surgical or fellow eye rejection transplantation, herpes simplex keratitis, and uncontrolled intraocular pressure (IOP), is the initial step in preventing immunological rejection. The primary method of preventing an immune graft response in all corneal grafts is the topical use of corticosteroids (39). Furthermore, FK506 eye drops, mycophenolate mofetil, and systemic cyclosporine A are effective in preventing rejection in high-risk penetrating keratoplasty (40). The local use of 0.1% tacrolimus significantly helps prevent corneal graft rejection in high-risk PKP patients. Additionally, reducing tacrolimus and corticosteroid levels effectively lowers the incidence of immune rejection after high-risk keratoplasty (41–43). This study found that timely local immune transplantation effectively controlled the disease. However, in cases where continuous immune inflammation led to graft dissolution, another PKP surgery was required. The study also showed that IVCM can detect rejection reactions early. In the future, identifying reliable biomarkers and establishing diagnostic scoring criteria will be necessary.

One of the main limitations of this study is that the data were collected from a tertiary referral center. This means that there may be limited available data. As a result of the study design, we could only obtain previously written reports and featured images, and we could not analyze confocal images or calculate measurable image features in all cases. Additionally, the immunotherapy treatment and duration for different cases may have been influenced by the physician’s experience and selection bias. This study included patients with heterogeneous ocular surface conditions prior to PK, which may have affected the generalizability of the findings. However, we aimed to explore whether most rejection cases could be related to preoperative pathogenic infections, such as fungal infections and corneal scarring from viral infections, providing a basis for further validation in future research.

## Conclusion

Both optical and therapeutic PKP can improve postoperative visual outcomes. In the event of a rejection reaction, the most apparent subjective symptom of the patient is the loss of visual acuity. Examination through IVCM suggests that the characteristic immune activation response of the endothelium is a marker of rejection after early PKP. Large implants and therapeutic PKP may be risk factors for the need for another PKP after rejection.

## Data Availability

The original contributions presented in the study are included in the article/supplementary material, further inquiries can be directed to the corresponding authors.
